# Development and application of base editing systems in *Salvia miltiorrhiza* for precise metabolic engineering

**DOI:** 10.1111/pbi.70234

**Published:** 2025-08-19

**Authors:** Qi Yao, Yi Ye, Qi Liu, Yifu Tian, Liu Xiang, Ling Li, Hang Liu, Yaojie Zhang, Muyao Yu, Han Zheng, Luqi Huang

**Affiliations:** ^1^ State Key Laboratory for Quality Ensurance and Sustainable Use of Dao‐di Herbs Nation al Resource Center for Chinese Materia Medica, China Academy of Chinese Medical Sciences Beijing China; ^2^ School of Agriculture and Biology Shanghai Jiao Tong University Shanghai China; ^3^ School of Ecological Technology and Engineering Shanghai Institute of Technology Shanghai China; ^4^ School of Traditional Chinese Medicine Beijing University of Chinese Medicine Beijing China

**Keywords:** *Salvia miltiorrhiza*, base editing, metabolic engineering, tanshinone, salvianolic acid

*Salvia miltiorrhiza* (*Danshen*), a key traditional medicinal plant for treating cardiovascular, inflammatory and oncological diseases (Zheng *et al*., 2023). While CRISPR/Cas‐mediated knockout enables gene disruptions in *Danshen*, precision base editing remains limited (Hsu *et al*., 2024). This study aimed to develop highly efficient adenine (A) and cytosine (C) base editors (ABEs and CBEs) for A‐to‐G or C‐to‐T conversions. By fusing a mutated N‐methylpurine DNA glycosylase (mMPG) to ABE, we further expand its editing capabilities to include A‐to‐T and A‐to‐C substitutions (Anzalone *et al*., 2020).

To investigate the feasibility of base editing in *Danshen*, we initially constructed first‐generation editors: SmCBEmax‐01 (CBE, using Arabidopsis‐optimized Anc689‐nCas9(D10A)‐UGI with AtU6‐driven sgRNAs) and SmABE8e‐01(ABE, using TadA8e‐nCas9(D10A) with AtU6‐driven sgRNAs and CaMV 35S promoter‐expressed editors) (Figure 1a, Figure S1). Seven sgRNAs targeting *SmMYB1*, *SmMYB36*, *SmMYB76*, *SmMYB39*, *SmbZIP2*, *SmJAZ3*, *SmbHLH3* (CBE) and five sgRNAs targeting *SmMYB1, SmC4H*, *SmKSL2*, *SmHMGR1*, *SmCPS2* (ABE) were designed (Figure 1b, Tables S1, S2) (Zheng *et al*., 2023). *Agrobacterium* (C58C1)‐mediated hairy root transformation generated 281 CBE and 397 ABE hygromycin‐resistant lines. Sanger sequencing revealed no detectable editing in CBE, while ABE exhibited 18.3% A‐to‐G editing efficiency (22/120 sequenced lines) at *SmMYB1*‐sgRNA2 A5 position. Limited efficiency was attributed to genome complexity, chromatin accessibility, and sgRNA/editor expression bottlenecks (Jiang *et al*., 2020). To optimize, we tested promoter combinations: native *SmU6* or *35SEN* (35S enhancer + CmYLCV + truncated U6‐26) for sgRNAs (Jiang *et al*., 2020), and endogenous *SmEF1α* or *SmRps5A* (shown to enhancing precision in other plants) for editors (Niu *et al*., 2022). This yielded second‐generation editors: SmCBEmax‐02/03 and SmABE8e‐02/03, with SmU6‐SmEF1α and 35SEN‐SmRps5A combinations, respectively (Figure 1a, Figure S1).

**Figure 1:**
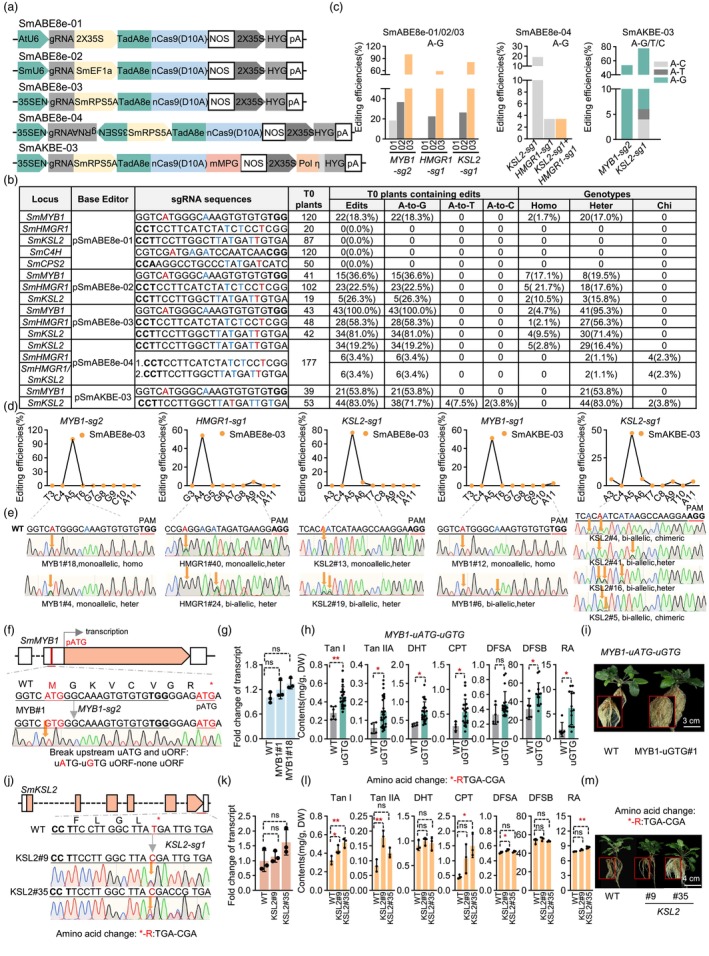
Development and application of adenine base editors in Salvia miltiorrhiza. (a) Base editors’ schematics. (b) Genotyping summary of T0 plants with ABEs. (c) A–G editing efficiencies across systems. (d) Editing windows of pSmABE8e‐03/pSmAKBE‐03. (e) Sanger sequencing of edited T0 lines (yellow arrows: Edited sites). (f) uATG/uORF disruption in SmMYB1 5’ UTR (A5‐G5). (j) SmKSL2 amino acid change (*252 TGA‐CGA, Arg/R). pATG: primary start codon; *: stop codon. (g, k) qPCR of SmMYB1 (g) and SmKSL2 (k) (mean ± SD, n = 3; normalized to WT; ns: not significant, one‐way ANOVA, P > 0.05). (h, l) Tanshinones/salvianolic acids in WT vs. edited lines, SmMYB1‐edited (h), and SmKSL2‐edited (l) (mean ± SD, n = 3; unpaired t‐test/h: P < 0.05; P < 0.01). (i, m) Phenotypic comparison of SmMYB1‐edited (i) and SmKSL2‐edited (m) lines with WT controls. (bold: PAM; red: target sites; blue: bystanders; ‘Homo’, ‘Heter’, ‘Chi’ denote homozygous, heterozygous, chimeric).

To rapidly assess the performance of optimized CBEs and ABEs, we selected *SmMYB1*‐sg1, *SmMYB76*‐sg1 and *SmMYB36*‐sg1 (CBE) and *SmMYB1*‐sg2, *SmKSL2*‐sg1 and *SmHMGR1*‐sg1 (ABE). Stable T0 plants (134 CBE, 295 ABE) were generated via *Agrobacterium tumefaciens* only 1 heterozygous C‐to‐T edit in CBEs (SmMYB36‐sg1, SmCBE‐max‐03; 1/72, 1.4%; Table S1, Figure S2). In contrast, optimized ABEs showed marked improvements: SmABE8e‐02 achieved 36.6%–22.5% A‐to‐G editing efficiencies at *SmMYB1*‐sg2, *SmHMGR1*‐sg1, *SmKSL2*‐sg1; while SmABE8e‐03 displayed 58.3%‐100.0% even with homozygous edits (Figure 1b,c). Further analysis of SmABE8e‐03 exhibited position‐dependent editing: A5 (100%) > A4 (58.3%); consecutive AA dinucleotides reduced editing (e.g. *SmKSL2*‐sg1: A5‐A6) showed reduced editing at the second adenine. Editing window spanned A4‐A9, with rare A9 conversion (unlike Arabidopsis/tomato) (Figure 1d,e) (Niu *et al*., 2022). To explore multiplex editing, SmABE8e‐03‐Dual (bidirectional sgRNAs) targeting *SmKSL2*‐sg1 and *SmHMGR1*‐sg1 was constructed (Figure 1a) (Fan *et al*., 2024). Among 177 transgenic plants, editing efficiencies decreased to 19.2% (34/177) and 3.4% (6/177) for individual targets, yet 3.4% (6/177) showed simultaneous editing, confirming feasibility (Figure 1b,c). These results validate SmABE8e‐03 as highly efficient for A•T‐to‐G•C base editing in *Danshen*.

MYB‐family transcription factors positively regulate their biosynthesis. Previous studies showed disrupting upstream ATG (uATG) or upstream open reading frames (uORFs) enhances protein translation efficiency through post‐translational regulation (Shen *et al*., 2024). We identified a 9‐amino‐acid uORF (MGKVCVGR*) in the 5′ UTR of *SmMYB1* and designed a target to convert uATG to uGTG, thereby disrupting both the uATG and uORF (Figure 1f). Using SmABE8e‐03, we obtained two homozygous A5‐to‐G5 conversions (Figure 1b). Transcript analysis showed no significant difference (Figure 1g) UPLC‐TQ‐MS revealed significant metabolic enhancements: tanshinone I/IIA, dihydrotanshinone I (1.6/2.5/1.9‐fold), cryptotanshinone, salvianolic acid A/B, rosmarinic acid (2.1/1.4/1.5/3.3‐fold) (Figure 1h). SmMYB‐uGTG lines exhibited normal growth despite metabolic boosts (Figure 1i).

To further expand editable base conversions (A•T‐to‐T•A/A•T‐to‐C•G) (Li *et al*., 2024), we constructed SmAKBE‐03 by fusing plant‐codon‐optimized mMPG to SmABE8e‐03 (Li *et al*., 2024; Wu *et al*., 2023), and endogenous SmPolη (incorporates AP sites to enhance A‐T editing) to the N‐terminus of HPTII (Figure 1a, Table S4). Evaluating SmAKBE‐03 at *SmMYB1*‐sg2 and *SmKSL2*‐sg1 via GV3101 transformation generated 39 and 53 transgenic lines. Sequencing revealed 71.7% (38/53) A‐to‐G, 7.5% (4/53) A‐to‐T, and 3.8% (2/53) A‐to‐C edits within the A3‐A11 window (PAM positions 21–23) for *SmKSL2*‐sg1; enables while *SmMYB1*‐sg2 achieved 53.8% (21/39) A‐to‐G efficiency with persistent A11 editing but no A‐to‐T/C products (Figure 1b,d). These findings confirm SmAKBE‐03 enables diversified base conversions in *Danshen*.

SmKSL1, catalyzing of copalyl diphosphate (CPP) into miltiradiene. Its homolog *SmKSL2* competitively depletes GGPP (CPP precursor), diverting metabolic flux (Zheng *et al*., 2023). The AKBE‐SmKSL2‐sg1 introduced A9‐to‐G9 substitution, converting the premature stop codon TGA (*252) to CGA (arginine, R), disrupting its structure (Figure 1j). Two edited lines harbored the A9‐G9 mutation (Figure 1b,j). Transcript analysis revealed no significant changes in *SmKSL2* expression (Figure 1k). UPLC‐TQ‐MS quantification demonstrated metabolite elevation: AKBE‐SmKSL2#9 increased tanshinone I/IIA, salvianolic acid A (1.4/2.3/1.1‐fold); AKBE‐SmKSL2#35 enhanced tanshinone I, cryptotanshinone, rosmarinic acid (1.6/3.2/1.1‐fold) (Figure 1l). Both lines maintained normal growth (Figure 1m). Metabolic variability likely stems from a downstream TGA (*254) preserving partial SmKSL2 protein integrity (Figure 1j). Though SmAKBE‐03 shows low A‐to‐T/C efficiencies and no A‐to‐Y edits in several sites (e.g. SmMYB1‐sg2), its broad editing window (A3‐A11) and A•T‐to‐T•A/C•G conversions highlight its potential.

To date, we have successfully established high‐efficiency ABE, ABE‐dual and AKBE editors in *Danshen*, surpassing prior systems with a more complete toolkit and improved efficiency (Han *et al*., 2024). SmABE8e‐03 achieves 100.0% editing efficiency and multiplex editing; while SmAKBE‐03 expanded the ABE window to A3‐11 and enables A‐to‐T/C conversion. Future, we will refine these systems for precise editing of key metabolic genes (e.g. *SmCPS1*, *SmCYP76AH1*), laying a robust foundation for next‐generation metabolic engineering in *S. miltiorrhiza*.

## Author contributions

Q.Y. and H.Z. proposed and designed the research; Q.Y., Y.Y. and Q.L. performed experiments; Q.Y. and L.L. analysed the data. X.L., H.L., M.Y., Y.Y., Y.T. and J.Z. performed transformation; Q.Y., H.Z. and L.‐Q.H. wrote and revised the manuscript. All authors read and approved the final manuscript.

## Supporting information


Figures S1–S2.
Tables S1–S4.

## Data Availability

All data generated or analysed during this study are included in this manuscript and its supplementary information files.
