# Supplementary material for: Development and application of base editing systems in Salvia miltiorrhiza for precise metabolic engineering
Source: Plant Biotechnol J. 2025 Aug 19;23(12):5619–21. doi: 10.1111/pbi.70234 (PMC12665085; doi:10.1111/pbi.70234)
Supplement: Supplementary file 1 — Figures S1–S2. Tables S1–S4. [file PBI-23-5619-s001.docx]

**Supplementary Information for**

**Development and application of base editing systems in *Salvia miltiorrhiza* for precise metabolic engineering**

**Supplementary Figures**

**Supplementary Figure 1.** Construction of the cytosine base editors.

**Supplementary Figure 2.** Editing results of C-T cytosine base editing.

**Supplementary Figure 3.** Phenotypes in of MYB1-uATG-uGTG editing lines.

**Supplementary Tables**

**Supplementary Table 1.** Summary of cytosine base editing results.

**Supplementary Table 2.** Base editing sgRNA target sites.

**Supplementary Table 3.** Primers used in this study.

**Supplementary Table 4.** DNA sequences of related vectors and genes.

**Supplementary Table 5.** Gene accession numbers in this study.

**
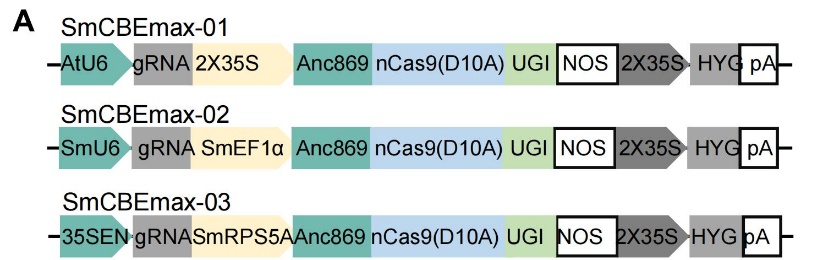
Supplemental Figure 1**. Construction of the cytosine base editors that we tried in this study.

**
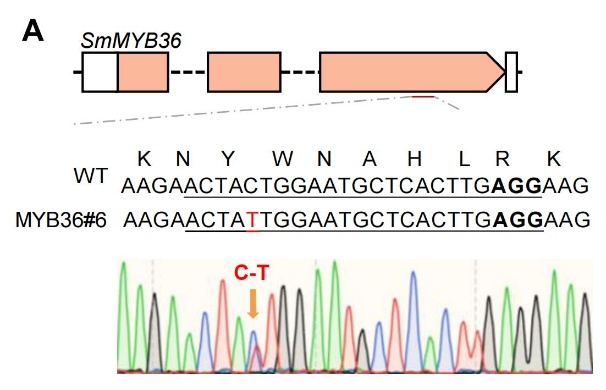
Supplemental Figure 2**. Cytosine base editing results of *SmMYB36* T0 lines. PAM and targeted base editing sites were marked in bold and red respectively, sgRNA was underlined.


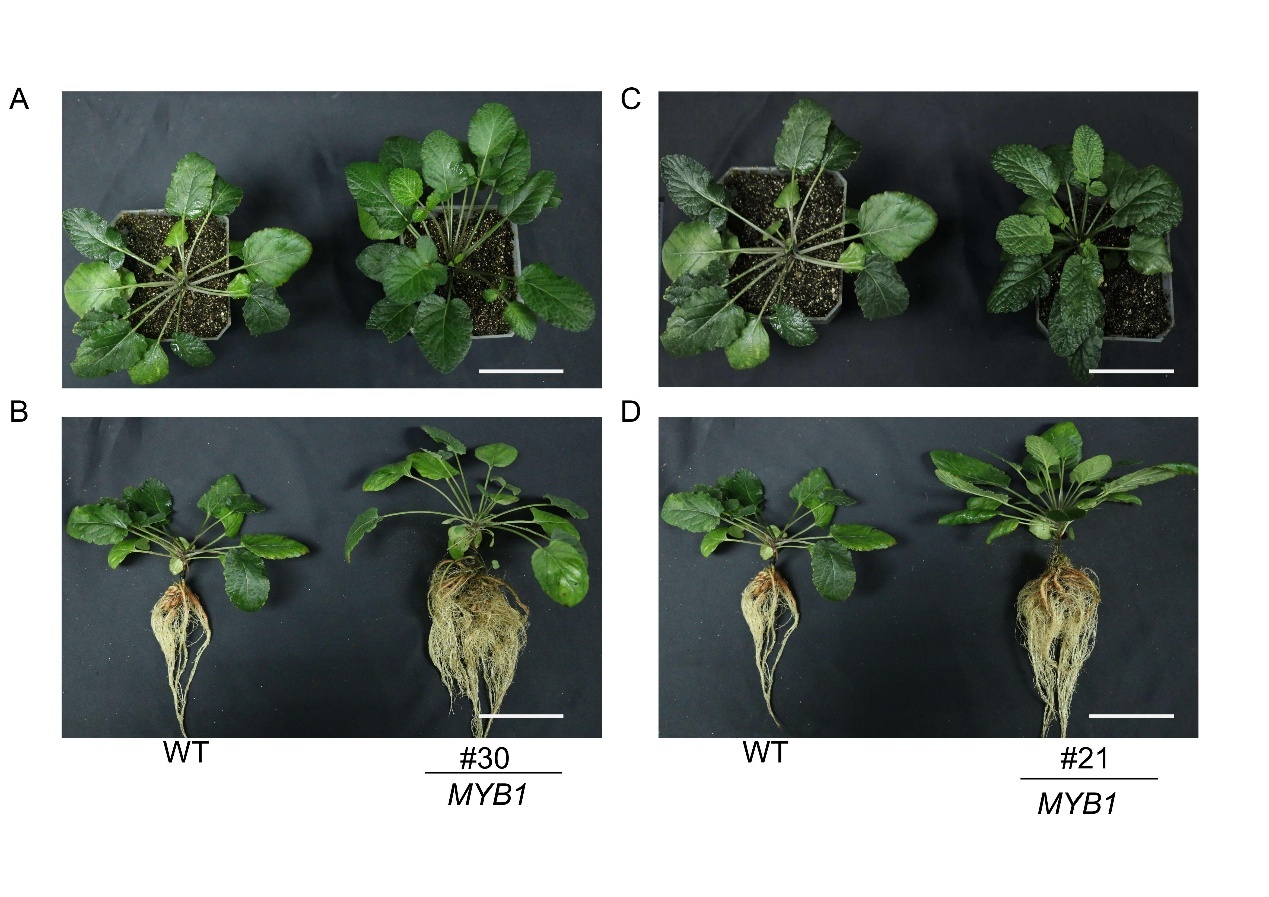


**Supplemental Figure 3**. Phenotypic comparison of SmMYB1-edited lines with WT controls. (A, C) aerial photograph of MYB1#30 and MYB1#21; (B, D) Root morphology of MYB1#30 and MYB1#21.

**Supplementary Table 1.** Summary of cytosine base editing results.

| **Targets** | **Editor** | **sgRNA or pegRNA sequence** | **T0 plants** | **T0 plants containing edits** | | **Genotype** | | |
| --- | --- | --- | --- | --- | --- | --- | --- | --- |
|  |  |  |  | Edits | C-to-T | Homo | Heter | Chi |
| *SmMYB1* | SmCBEmax-01 | CCAAGCGTCTAGGGAAGTGGTGG | 55 | 0 | 0 | 0 | 0 | 0 |
| *SmMYB76* |  | CCACGGCGAGGGATGCTGGCGCT | 54 | 0 | 0 | 0 | 0 | 0 |
| *SmMYB36* |  | ACTACTGGAATGCTCACTTG**AGG** | 26 | 0 | 0 | 0 | 0 | 0 |
| *SmbZIP2* |  | CTGCTGCAGCCAACAATGCC**GGG** | 37 | 0 | 0 | 0 | 0 | 0 |
| *SmJAZ3* |  | TCCTACGCTGCTCCTGTGGC**CGG** | 36 | 0 | 0 | 0 | 0 | 0 |
| *SmMYB39* |  | GAGCGCCCGACTCCAAGCCG**AGG** | 73 | 0 | 0 | 0 | 0 | 0 |
| *SmbHLH3* |  | GCTCTACGATTATGGCTTCG**AGG** | 44 | 0 | 0 | 0 | 0 | 0 |
| *SmMYB1* | SmCBEmax-02 | **CCA**AGCGTCTAGGGAAGTGGTGG | 20 | 0 | 0 | 0 | 0 | 0 |
| *SmMYB76* |  | **CCA**CGGCGAGGGATGCTGGCGCT | 19 | 0 | 0 | 0 | 0 | 0 |
| *SmMYB36* |  | ACTACTGGAATGCTCACTTG**AGG** | 23 | 0 | 0 | 0 | 0 | 0 |
| *SmMYB1* | SmCBEmax-03 | **CCA**AGCGTCTAGGGAAGTGGTGG | 20 | 0 | 0 | 0 | 0 | 0 |
| *SmMYB36* |  | ACTACTGGAATGCTCACTTG**AGG** | 72 | 1 | 1 | 0 | 1 | 0 |
| Note: PAM and targeted base editing sites were marked in bold and red respectively, potential bystander editing sites were marked in blue. “Homo”, homozygous; “Heter”, heterozygous; “Chi”, chimeric. | | | | | | | | |

**Supplementary Table 2.** Base editing sgRNA target sites.

| **Name** | **Methods** | **Editing results** | **Target sequence (5'-3')** | **Position to 5'** |
| --- | --- | --- | --- | --- |
| SmMYB1-sgRNA1 | CBE | C-T | **CCA**AGCGTCTAGGGAAGTGGTGG | -4 |
| SmMYB76-sgRNA1 |  | C-T | **CCA**CGGCGAGGGATGCTGGCGCT | -5 |
| SmbHLH3-sgRNA1 |  | C-T | GCTCTACGATTATGGCTTCG**AGG** | -7 |
| SmMYB36-sgRNA1 |  | C-T | ACTACTGGAATGCTCACTTG**AGG** | -5 |
| SmMYB39-sgRNA1 |  | C-T | GAGCGCCCGACTCCAAGCCG**AGG** | -4 |
| SmbZIP2-sgRNA1 |  | C-T | CTGCTGCAGCCAACAATGCC**GGG** | -4 |
| SmJAZ3-sgRNA1 |  | C-T | TCCTACGCTGCTCCTGTGGC**CGG** | -6 |
| SmMYB1-sgRNA2 | ABE | A-G | GGTCATGGGCAAAGTGTGTG**TGG** | -5 |
| SmC4H-sgRNA1 |  |  | CGTCGATGAGATCCAATCAA**CGG** | -6 |
| SmKSL2-sgRNA1 |  |  | **CCT**TCCTTGGCTTATGATTGTGA | -5 |
| SmHMGR1-sgRNA1 |  |  | **CCT**CCTTCATCTATCTCCTCGG | -4 |
| SmGRAS1-sgRNA1 |  |  | **CCA**CTACAACCCCATTTCATGGC | -4 |
| SmCPS4-sgRNA1 |  |  | GATAGTGATGACACCTCCAT**GGG** | -4 |
| SmMYC2a-sgRNA1 |  |  | CTAAAGAGGCGCAGATCCAC**GGG** | -3 |
| SmMYB1-sgRNA2 | AKBE | A-T/C/G | GGTCATGGGCAAAGTGTGTG**TGG** | -11 |
| SmKSL2-sgRNA1 |  |  | **CCT**TCCTTGGCTTATGATTGTGA | -9 |
| SmHMGR1-sgRNA1 | ABE Dual | A-G | **CCT**CCTTCATCTATCTCCTCGG | -4 |
| SmKSL2-sgRNA1 |  |  | **CCT**TCCTTGGCTTATGATTGTGA | -5 |
| Note: PAM and targeted base editing sites were marked in bold and red respectively, potential bystander editing sites were marked in blue. | | | | |

**Supplementary Table 3.** Primers used in this study.

| **Primer name** | **Sequence (5'-3')** | **Experiment** |
| --- | --- | --- |
| CaMv35S-F | CCACGTCTTCAAAGCAAG | Amplification of positive transgene target sites |
| CaMv35S-R | TCCTCTCCAAATGAAATGAACTTCC |  |
| HPT-F | AGCTGCGCCGATGGTTTCTACAA |  |
| HPT-R | ATCGCCTCGCTCCAGTCAATG |  |
| Cas9-F1 | GAGTACAAGGTGCCCTCAAAG |  |
| Cas9-R1 | TCCTCAGCGAGATCGAAATT |  |
| Kan-F | CAAGATGGATTGCACGCAGG |  |
| Kan-R | CGCTATGTCCTGATAGCGGT |  |
| SmMYB1-CX-F | GGTGGGTGCACATGAATTTT | Amplification of base editing sample results. |
| SmMYB1-CX-R | GACGAGAGACTGCTCCTCTTGG |  |
| SmMYB1-CX-F1 | CCAAGAGGAGCAGTCTCTCGTC |  |
| SmMYB1-CX-R1 | TGCTTCAAGCTGCAGCTCC |  |
| SmHMGR1-CX-F | CCGTCGTCTACTTTCTCCTCCT |  |
| SmHMGR1-CX-R | AATCTGCACGTACCCGAC |  |
| SmHMGR1-CX-F2 | CCGTCGTCTACTTTCTCCTCCT |  |
| SmHMGR1-CX-R2 | AATCTGACGTACCCGAC |  |
| SmC4H-CX-F | CGACTCGACACTCACGATTCG |  |
| SmC4H-CX-R | CATTATCCTTGGCCGCGAT |  |
| SmKSL2-CX-F | ATGGACGGAGGGAGTACCAA |  |
| SmKSL2-CX-R | GACCGTGAAACAAATCTACTTGAA |  |
| SmMYB76-CX-F | TGATCGAGTGCGATGGGAAG |  |
| SmMYB76-CX-R | GGATGCCGAGACTACAAGCG |  |
| SmbHLH3-CX-F1 | CGAAGACCTCCTTTTTCCCTGC |  |
| SmbHLH3-CX-R1 | AAGAGCACCTGGCATTGGTC |  |
| SmbHLH3-KZ-F1 | CCAAGGCGATGCAAATCATCC |  |
| SmbHLH3-KZ-R1 | CGGTCTCTAGTCCAACATTATCC |  |
| SmMYB36-CX-F1 | ATGAATAGAGATGGCGAGTGATGC |  |
| SmMYB36-CX-R1 | GCTGGTGATGAATTGCAGCAT |  |
| SmMYB36-CX-F1 | ATGAATAGAGATGGCGAGTGATGC |  |
| SmMYB36-CX-jc-R3 | GGATTCATCGTCGCCTCCG |  |
| SmMYB39-CX-F1 | GGTCCACCATAGCAAGTCAG |  |
| SmMYB39-CX-R1 | CTCACGACCCACAAACATCA |  |
| SmMYB76-CX-F1 | TGATCGAGTGCGATGGGAAG |  |
| SmMYB76-CX-R1 | GGATGCCGAGACTACAAGCG |  |
| SmJAZ3-CX-F1 | GCTTACCAATGCAGTGGTCAT |  |
| SmJAZ3-CX-R1 | GGGAGAAATGTCGTCGTACACG |  |
| SmbZIP2-CX-F2 | TGGAGGGATATGTCGAAGGAG |  |
| SmbZIP2-CX-R2-1 | GCTGGTGTTGGTGTTGATGT |  |
| SmbHLH3-CX-F1 | CGAAGACCTCCTTTTTCCCTGC |  |
| SmbHLH3-CX-R1 | AAGAGCACCTGGCATTGGTC |  |
| SmMYB36-CX-F1 | ATGAATAGAGATGGCGAGTGATGC |  |
| SmMYB36-CX-jc-R3 | GGATTCATCGTCGCCTCCG |  |
| SmMYB39-CX-F1 | GGTCCACCATAGCAAGTCAG |  |
| SmMYB39-CX-R1 | CTCACGACCCACAAACATCA |  |
| SmbZIP2-CX-F2 | TGGAGGGATATGTCGAAGGAG |  |

**Supplementary Table 3.** Continued.

| **Primer name** | **Sequence (5'-3')** | **Experiment** |
| --- | --- | --- |
| SmbZIP2-CX-R2-1 | GCTGGTGTTGGTGTTGATGT | Amplification of base editing sample results. |
| SmbHLH3-CX-F1 | CGAAGACCTCCTTTTTCCCTGC |  |
| SmbHLH3-CX-R1 | AAGAGCACCTGGCATTGGTC |  |
| SmMYB36-CX-F1 | ATGAATAGAGATGGCGAGTGATGC |  |
| SmMYB36-CX-jc-R3 | GGATTCATCGTCGCCTCCG |  |
| SmMYB39-CX-F1 | GGTCCACCATAGCAAGTCAG |  |
| SmMYB39-CX-R1 | CTCACGACCCACAAACATCA |  |
| SmbZIP2-CX-F2 | TGGAGGGATATGTCGAAGGAG |  |
| SmbZIP2-CX-R2-1 | GCTGGTGTTGGTGTTGATGT |  |
| SmMYC2a-F1 | CCACTCCGACTCCGACTTGTTC |  |
| SmMYC2a-R1 | CTTGTCCGACTGCACCCTGAT |  |

**Supplementary Table 4.** DNA sequences of related vectors and genes.

| >SmU6 promoter ATTTACTGTTCACGACCGTTAGTAAATAAAATATGATATAAGTTGTGGATTTTGTATGAAATAATATGTAAGAAAATGAAGCATGTTGTTGTATATGTAAAAGAATGTGTGCAGATGTGCATGGGGCAATTAGCAACAGCTTGGCATTTGATGATGAATGTGCGTTTGTTTCTCTATTTCCGACTGGCAATGTCCATTGCAACCCCACTATATATTTAATTAATTTGTCACTAACAATATATAATTAAAAACCTATGCATATATACACATTAAGAATGGTTTCAAATGAGTTAAGGTATATATTCTATAAAATAGTGTAGTTATTTTCAGTCTTTTAATGTGAATATAAATTTAAAGTAGATTCATCACTTTGTTGAATAAAGTAAATAAATCTGATTTTTATTATAATCGACTCTACTTTATCTCTACTTTATCCAGGCACACATACACATCTATGTGTATGTATATGTATATATATATTGTCAAATCAATATCGACCTTAAAAAGTATGGACCTCCGACTTATATATATTACTATCAAATTATTATCATAATATAATCAATTATATTATGGTATATATCGATTTGCCACCTATACTTCTGAAATTTGTACATGCAGGTTGAAACCTCGACTTCCCATTTATAGGAGGTGAGATTCCATTTGTTAAGTTTCAAAGTTCTAGTTGTTTTCTTTAATCTACACCTATTGAAAAATAATTAAAAAATCGAGTCAAATCAAACCAAATTAAAATTTATCTTTAGTTAGTTACTAGCATGTTGTTGAATATAAATGTAGGATTCCATTTGTTAAGTTTCAAAGTTCTAGTTGTTTTCTTTAATCTACACCTATTGAAAAATCATTAAAATCGAGTCAAATCAAACCAAATTAAAATTTATCTTTTTTTTCTTCTTTATAAAACCAAATATTTATCTTTAGTTACTAGCATGTTGTTGATATTCATATTTATAGGATAAATTCTAGTATGAAAATATAGGCTCAGACAATATTTACAATTCGGGTTAAAAGATCAGACCATAAGACATATATAATTAACTATATATAGATGTGTAGAAAGAATAAAAATTAGTAATAAACTTCACTTAATTATCGTAAAAAGAATATCATAACAAAAAATAATTGTATAAGAAAATATAATTTTATATTGAAAACTGAAATTAAAAGCTTAAAACTTGTCGTAAAATATGCTGTAAACATATTTTAATCATATGATAATGTGTGACAATGTCCATAAACTTGTTTTAAGCATATCTGACATTTTGTTCCATTTTCTCTTAAACTTATTAATTTTGTGTTGGGTATTCCAAATTTAATGTATTTATAATGAAGTAGTATAAAATTCAATTGGAATTGGGCCAAGATATAACAGCTCTAAGCCCAACCCAAAGCCCACACTGTTTTCAAAACCAAGTTTTCATCCCACATCGAATTCCAATGGGTTTCTTAAGCGTTTTATATACCTCCATTCTCTATGTATATATTGTCCCTTCGGGGACATCCGATAAAATTGGAACGATACAGAGAAGATTAGCATGGCCCCTGCGCAAGGATGACACGCATAAATCGAGAAATGGTCCAAATTTTTTTGTCAA |
| --- |
| >35SEN promoter,35SE-CmYLCV-SmU6-26 ATGGAGTCAAAGATTCAAATAGAGGACCTAACAGAACTCGCCGTAAAGACTGGCGAACAGTTCATACAGAGTCTCTTACGACTCAATGACAAGAAGAAAATCTTCGTCAACATGGTGGAGCACGACACACTTGTCTACTCCAAAAATATCAAAGATACAGTCTCAGAAGACCAAAGGGCAATTGAGACTTTTCAACAAAGGGTAATATCCGGAAACCTCCTCGGATTCCATTGCCCAGCTATCTGTCACTTTATTGTGAAGATAGTGGAAAAGGAAGGTGGCTCCTACAAATGCCATCATTGCGATAAAGGAAAGGCCATCGTTGAAGATGCCTCTGCCGACAGTGGTCCCAAAGATGGACCCCCACCCACGAGGAGCATCGTGGAAAAAGAAGACGTTCCAACCACGTCTTCAAAGCAAGTGGATTGATGTGATTGGCAGACATACTGTCCCACAAATGAAGATGGAATCTGTAAAAGAAAACGCGTGAAATAATGCGTCTGACAAAGGTTAGGTCGGCTGCCTTTAATCAATACCAAAGTGGTCCCTACCACGATGGAAAAACTGTGCAGTCGGTTTGGCTTTTTCTGACGAACAAATAAGATTCGTGGCCGACAGGTGGGGGTCCACCATGTGAAGGCATCTTCAGACTCCAATAATGGAGCAATGACGTAAGGGCTTACGAAATAAGTAAGGGTAGTTTGGGAAATGTCCACTCACCCGTCAGTCTATAAATACTTAGCCCCTCCCTCATTGTTAAGGGAGCAAAATCTCAGAGAGATAGTCCTAGAGAGAGAAAGAGAGCAAGTAGCCTAGAAGTAGTCAAGGCGGCGAAGTATTCAGGCACGTGGCCAGGAAGAAGAAAAGCCAAGACGACGAAAACAGGTAAGAGCTAAGCTGTATATATTGTCCCTTCGGGGACATCCGATAAAATTGGAACGATACAGAGAAGATTAGCATGGCCCCTGCGCAAGGATGACACGCATAAATCGAGAAATGGTCCAAATTTTTTTGTCAA |
| >SmEF1α promoter GAAATAAATTAGTGTGAATAGCAAAGTAGAAATACAATTACTCAGTGATTCATACGTAGGGAAATCGCGTGAAAAAACTGTATAACATATATATTTTTTGAATGTGTTACATTTTTTTGTGGAGAATGAGAATATCTAAATTAGTGAAAACAAATCTATGATTATTTTTAGAAGAATCACCAAATATATTGGAAACAATATATTGAGTATATTTATCAACGGATAATTAGTGGAAACAAATAAAAATAACAAAATTTTAGCAATATAAATATCCGAAATAATAACATAATTTAATATTAGATAAAAATTTATTTTTATCTTATAAAAAAATCTAATTTTCAAAAAATTAAAAAATAATATCCGAAAACAAATCTAGTACTTATTTTTTTAAGGAGAAACACAAAATCAGTTGAAACAAATTAATAAAAAACAAATAAACACAAAATTTAGGAGTAATAATTTTTGAAAAATAAATAAATAATATTAATTTAGAAAAAAAAATATTCGGAAACAAATCTAGAATTATTATAACAAGAAACACTAAATTATTGGTAACAAATCAATAATAAAATTTAAAAACAAATAAATCAATAACAAAACCGAAAAAAAAATATTCGAATTATATAATTTATAAAATTTTCTGAAATAAAAACATAAAAATAAGGAAACAAAGTCTTCAAAAATTAAGGAAACAAAATGGAAGATTTTGTGAAAATTAGAAACAAATTAGCCGAAAAAAAAGGAAAGAAAATGGACGAAAAATAAGGAAAGAGAATGGACGAAAAAATCAAGGGAAAGAGACTAATTTCAGATATATATACATGGAAAAAATCAAGGGATAAAACAGTAAAACCAATAAATCCCTAAAACCGGCCCACTCTCTACCCACCAAAAAAATCGATTTTTTACTAGGTGTTCAGTACACCTGGTGTCCGTTTATCACTACTCTTTAATAAAATGTGAGTGAAGTTGGTCCAGTAAGGATCCCACTTTAAATATGAGTGGAATGATGTGGACCCTATTACTAAAACTAAAAATGAATGTGACATATTTTTTGTGGACGGACGAAAAATGAAATTGTGGCATATCTTTGGTGGACGGGGGAGTACTATTGTTTTAATCAATTTTATCGAGAAGGATATATTGTTAATCGTTGCCCTAATTAAAAGAAGCGTGGACTTTCTATATACTTGGATCCCAAGTCAAAGAAGGACTGCACAGGCAGACTCTCACAGGGGTATAACAGTCAATCCGATGAAAATATATCTCCCTAAACCCTAGCTGCTTAGTATAAATACGTGCACTCCCCACCTCTCTAAACACTCTGCACCTCCGCTCTCCTTCCCCAATTACATCTTGTTTGTTCTTTGAAGGTAAAAATTGCTCTACCCGTTCTAATATGCTTCGATNTTTCTTGTACTCGTAGGATTTTGTAGCTGTTGATTTGTTGTTATATTTTTGTCGATTTGATTTATGTAAAGCGCTTTGTTTGTGGAAAAATTTCATTGAATTTTCAGATCTATCTGCTTGTTTATGAATTAATAGCTCACTCCCAATGAATTTTTACGATCCTTAAATCTCTGTTGGAGCATATGATTAATGTATTATTGTTTTGCTCTCTGGTCGGTTGAGGTCTGATTTACTTAGTTGATGAATCGTATTCGTGGTTAAGCATGTTTGAGTGAGTCTCTGTATAACTTTAATGGTAGTGTAGTTGTATCGGCATGCTCTGAAACGATTTGTCTAGTTTCATTGGTCATTCGTATATTTATATAAGGTATTCGTCCCGTTTCTCAAGCTGTTTGAGATGTATATGATACTTTGCGAATTAATGACTTTAAGTTTTTTACTTTTTTTATTTTCCTAGATCTATAATTTAGATTTTCATATCTTTTGCTAAAGAAGCTATGAGTCGTATGATAAGCTTCATGTTATGTTATATAACATCATTGTTTGATTCTTATTTGTTGCAGGTTTATTTATCTCTTAACAGTTGAAAT |
| >SmRPS5A TAAGCTTAATAATACTCATTTCGTTCATGAAAATTATAGCATTATTACTTCCTCCGTTTCATTTATAACGTTCCAAAAAAATTGAAATAGGCAAATTAAAGAAATATAAATAAAGTAAATGATGAGAGAGTTAGAGAAAATAGTGAAAAAATAAATAAAGATAAAGTAAACAAGGAGAGAGATAAAGAGAGTAGATAAAGTAAAAAAAAAAAAAATTGAAGCTACCCCTTATATTTTAAATTTCATTTACACCCAAATGTACAAAATACCCAACAATTTATTGTGCATCAATTTTCATGAGTCTTTTTTTTTTTATCTTTGTACATATTTTTCAACTATTTTTAAAATTCGTTTCCTCAAACTCACTACAATTTTCGTGAACGGGACGAAATGAAGAGAATACAACATAGGGTGATTCTATTCATAGCTTCCATTTTACTCTTTACAGCTCCTTTTTAAGATATTAATAATAATTAATTAAAAATTTACTATTTTATCATATGTTGTATAGTCCCCAAATTATATTAAATTTTAAATTAATTCATTACTTCAATTTCTTTTTACTATTTACTTTTATCTTAATATTGTGTATTCGTTTGTAGCCCACATTTGCCACCGCCCCAGAGTTCCTCCGCTGCCAGTGATGGCGCCGCCGACCAGCTGACCCTTTCCTTTCAGGTAAAGTTTTTTTTTTTTTTGTAATTTTTGGCCGAATCATCAAGGAAAGAGATGTTGAGATAGTCCTTGTATTGTAGGTGGAGGTGTTACTCTCCTCTACGCCGCCAAAGAACTGGAGAAGCTGCCAACAACCAACTTTGATCAGAAAAATGGTGTCCAAATTATTCAAAATGCTTTGAAGGTTTGATTATGACAATATATGCTCCATTATAGCAATAGCGCTTGATATGTTATTATTATTAATATTTTAACAAGGGTCTATAAGGTGTAAGGAGTAAAATAGGGGCTCTGAATGACCCTAGTGTTATTGTTTGAATGGACTGTAATTTTGAAGAGGCCCATCACCCTTTTAGCATAAGGAGAATGAACAAACGGGCTAAAGCGTGAATTGAGGCCCAAACCCACAACTCCTATTAAAACTGCCATTAGGGTTTCGGCCCCTCTTTCTTCACCAATACTTCCATTCAGCTTCCCGCACACTGCAACAACCATACGCCGTCGATATTCCTCAAGGTTTACTCTCTCTATCTGCCTTCCTCTCTAGTATTGCTTTCTGTATGCTCATAAGTATCAATTTCAGGTGAATCGCGGAGAAGAT |
| >SmABE8e-01/02/03/SmABE8e-03-Dual,NLS-TadA8e(V106W)-linker-nCas9-NLS ATGAAACGGACAGCCGACGGAAGCGAGTTCGAGTCACCAAAGAAGAAGCGGAAAGTCTCTGAGGTGGAGTTCAGCCACGAGTACTGGATGAGGCACGCCCTGACCCTGGCAAAGCGGGCCAGAGACGAGAGAGAGGTGCCCGTGGGAGCCGTGCTGGTGCTGAACAATAGAGTGATCGGCGAGGGCTGGAACAGAGCCATCGGCCTGCACGACCCTACAGCACACGCAGAGATCATGGCACTGAGGCAGGGAGGCCTGGTCATGCAGAATTACCGCCTGATCGATGCCACCCTGTATGTGACATTCGAGCCATGCGTGATGTGCGCAGGAGCAATGATCCACAGCAGGATCGGCCGCGTGGTGTTTGGATGGAGGAACTCCAAGAGGGGAGCAGCAGGCTCTCTGATGAACGTGCTGAATTACCCAGGCATGAATCACCGGGTGGAGATCACCGAGGGCATCCTGGCAGACGAGTGCGCCGCCCTGCTGTGCGATTTCTATCGGATGCCCAGACAGGTGTTTAACGCCCAGAAGAAGGCCCAGAGCAGCATCAACTCCGGAGGATCTAGCGGAGGCTCCTCTGGCTCTGAGACACCTGGCACAAGCGAGAGCGCAACACCTGAAAGCAGCGGGGGCAGCAGCGGGGGATCCGACAAGAAGTACAGCATCGGCCTGGCCATCGGCACCAACTCTGTGGGCTGGGCCGTGATCACCGACGAGTACAAGGTGCCCAGCAAGAAATTCAAGGTGCTGGGCAACACCGACCGGCACAGCATCAAGAAGAACCTGATCGGAGCCCTGCTGTTCGACAGCGGCGAAACAGCCGAGGCCACCCGGCTGAAGAGAACCGCCAGAAGAAGATACACCAGACGGAAGAACCGGATCTGCTATCTGCAAGAGATCTTCAGCAACGAGATGGCCAAGGTGGACGACAGCTTCTTCCACAGACTGGAAGAGTCCTTCCTGGTGGAAGAGGATAAGAAGCACGAGCGGCACCCCATCTTCGGCAACA  TCGTGGACGAGGTGGCCTACCACGAGAAGTACCCCACCATCTACCACCTGAGAAAGAAACTGGTGGACAGCACCGACAAGGCCGA  CCTGCGGCTGATCTATCTGGCCCTGGCCCACATGATCAAGTTCCGGGGCCACTTCCTGATCGAGGGCGACCTGAACCCCGACAACAGCGACGTGGACAAGCTGTTCATCCAGCTGGTGCAGACCTACAACCAGCTGTTCGAGGAAAACCCCATCAACGCCAGCGGCGTGGACGCCAAGGCCATCCTGTCTGCCAGACTGAGCAAGAGCAGACGGCTGGAAAATCTGATCGCCCAGCTGCCCGGCGAGAAGAAGAATGGCCTGTTCGGAAACCTGATTGCCCTGAGCCTGGGCCTGACCCCCAACTTCAAGAGCAACTTCGACCTGGCCGAGGATGCCAAACTGCAGCTGAGCAAGGACACCTACGACGACGACCTGGACAACCTGCTGGCCCAGATCGGCGACCAGTACGCCGACCTGTTTCTGGCCGCCAAGAACCTGTCCGACGCCATCCTGCTGAGCGACATCCTGAGAGTGAACACCGAGATCACCAAGGCCCCCCTGAGCGCCTCTATGATCAAGAGATACGACGAGCACCACCAGGACCTGACCCTGCTGAAAGCTCTCGTGCGGCAGCAGCTGCCTGAGAAGTACAAAGAGATTTTCTTCGACCAGAGCAAGAACGGCTACGCCGGCTACATTGACGGCGGAGCCAGCCAGGAAGAGTTCTACAAGTTCATCAAGCCCATCCTGGAAAAGATGGACGGCACCGAGGAACTGCTCGTGAAGCTGAACAGAGAGGACCTGCTGCGGAAGCAGCGGACCTTCGACAACGGCAGCATCCCCCACCAGATCCACCTGGGAGAGCTGCACGCCATTCTGCGGCGGCAGGAAGATTTTTACCCATTCCTGAAGGACAACCGGGAAAAGATCGAGAAGATCCTGACCTTCCGCATCCCCTACTACGTGGGCCCTCTGGCCAGGGGAAACAGCAGATTCGCCTGGATGACCAGAAAGAGCGAGGAAACCATCACCCCCTGGAACTTCGAGGAAGTGGTGGACAAGGGCGCTTCCGCCCAGAGCTTCATCGAGCGGATGACCAACTTCGATAAGAACCTGCCCAACGAGAAGGTGCTGCCCAAGCACAGCCTGCTGTACGAGTACTTCACCGTGTATAACGAGCTGACCAAAGTGAAATACGTGACCGAGGGAATGAGAAAGCCCGCCTTCCTGAGCGGCGAGCAGAAAAAGGCCATCGTGGACCTGCTGTTCAAGACCAACCGGAAAGTGACCGTGAAGCAGCTGAAAGAGGACTACTTCAAGAAAATCGAGTGCTTCGACTCCGTGGAAATCTCCGGCGTGGAAGATCGGTTCAACGCCTCCCTGGGCACATACCACGATCTGCTGAAAATTATCAAGGACAAGGACTTCCTGGACAATGAGGAAAACGAGGACATTCTGGAAGATATCGTGCTGACCCTGACACTGTTTGAGGACAGAGAGATGATCGAGGAACGGCTGAAAACCTATGCCCACCTGTTCGACGACAAAGTGATGAAGCAGCTGAAGCGGCGGAGATACACCGGCTGGGGCAGGCTGAGCCGGAAGCTGATCAACGGCATCCGGGACAAGCAGTCCGGCAAGACAATCCTGGATTTCCTGAAGTCCGACGGCTTCGCCAACAGAAACTTCATGCAGCTGATCCACGACGACAGCCTGACCTTTAAAGAGGACATCCAGAAAGCCCAGGTGTCCGGCCAGGGCGATAGCCTGCACGAGCACATTGCCAATCTGGCCGGCAGCCCCGCCATTAAGAAGGGCATCCTGCAGACAGTGAAGGTGGTGGACGAGCTCGTGAAAGTGATGGGCCGGCACAAGCCCGAGAACATCGTGATCGAAATGGCCAGAGAGAACCAGACCACCCAGAAGGGACAGAAGAACAGCCGCGAGAGAATGAAGCGGATCGAAGAGGGCATCAAAGAGCTGGGCAGCCAGATCCTGAAAGAACACCCCGTGGAAAACACCCAGCTGCAGAACGAGAAGCTGTACCTGTACTACCTGCAGAATGGGCGGGATATGTACGTGGACCAGGAACTGGACATCAACCGGCTGTCCGACTACGATGTGGACCATATCGTGCCTCAGAGCTTTCTGAAGGACGACTCCATCGACAACAAGGTGCTGACCAGAAGCGACAAGAACCGGGGCAAGAGCGACAACGTGCCCTCCGAAGAGGTCGTGAAGAAGATGAAGAACTACTGGCGGCAGCTGCTGAACGCCAAGCTGATTACCCAGAGAAAGTTCGACAATCTGACCAAGGCCGAGAGAGGCGGCCTGAGCGAACTGGATAAGGCCGGCTTCATCAAGAGACAGCTGGTGGAAACCCGGCAGATCACAAAGCACGTGGCACAGATCCTGGACTCCCGGATGAACACTAAGTACGACGAGAATGACAAGCTGATCCGGGAAGTGAAAGTGATCACCCTGAAGTCCAAGCTGGTGTCCGATTTCCGGAAGGATTTCCAGTTTTACAAAGTGCGCGAGATCAACAACTACCACCACGCCCACGACGCCTACCTGAACGCCGTCGTGGGAACCGCCCTGATCAAAAAGTACCCTAAGCTGGAAAGCGAGTTCGTGTACGGCGACTACAAGGTGTACGACGTGCGGAAGATGATCGCCAAGAGCGAGCAGGAAATCGGCAAGGCTACCGCCAAGTACTTCTTCTACAGCAACATCATGAACTTTTTCAAGACCGAGATTACCCTGGCCAACGGCGAGATCCGGAAGCGGCCTCTGATCGAGACAAACGGCGAAACCGGGGAGATCGTGTGGGATAAGGGCCGGGATTTTGCCACCGTGCGGAAAGTGCTGAGCATGCCCCAAGTGAATATCGTGAAAAAGACCGAGGTGCAGACAGGCGGCTTCAGCAAAGAGTCTATCCTGCCCAAGAGGAACAGCGATAAGCTGATCGCCAGAAAGAAGGACTGGGACCCTAAGAAGTACGGCGGCTTCGACAGCCCCACCGTGGCCTATTCTGTGCTGGTGGTGGCCAAAGTGGAAAAGGGCAAGTCCAAGAAACTGAAGAGTGTGAAAGAGCTGCTGGGGATCACCATCATGGAAAGAAGCAGCTTCGAGAAGAATCCCATCGACTTTCTGGAAGCCAAGGGCTACAAAGAAGTGAAAAAGGACCTGATCATCAAGCTGCCTAAGTACTCCCTGTTCGAGCTGGAAAACGGCCGGAAGAGAATGCTGGCCTCTGCCGGCGAACTGCAGAAGGGAAACGAACTGGCCCTGCCCTCCAAATATGTGAACTTCCTGTACCTGGCCAGCCACTATGAGAAGCTGAAGGGCTCCCCCGAGGATAATGAGCAGAAACAGCTGTTTGTGGAACAGCACAAGCACTACCTGGACGAGATCATCGAGCAGATCAGCGAGTTCTCCAAGAGAGTGATCCTGGCCGACGCTAATCTGGACAAAGTGCTGTCCGCCTACAACAAGCACCGGGATAAGCCCATCAGAGAGCAGGCCGAGAATATCATCCACCTGTTTACCCTGACCAATCTGGGAGCCCCTGCCGCCTTCAAGTACTTTGACACCACCATCGACCGGAAGAGGTACACCAGCACCAAAGAGGTGCTGGACGCCACCCTGATCCACCAGAGCATCACCGGCCTGTACGAGACACGGATCGACCTGTCTCAGCTGGGAGGCGACAAAAGAACCGCCGACGGCAGCGAATTCGAGCCCAAGAAGAAGAGGAAAGTC |
| >SmAYBE-03, NLS-TadA8e(V106W)-linker-nCas9-NLS-linker-MPG-biNLS-Danshen TLS polymerase *η*-HYG-polyA  ATGAAACGGACAGCCGACGGAAGCGAGTTCGAGTCACCAAAGAAGAAGCGGAAAGTCTCTGAGGTGGAGTTCAGCCACGAGTACTGGATGAGGCACGCCCTGACCCTGGCAAAGCGGGCCAGAGACGAGAGAGAGGTGCCCGTGGGAGCCGTGCTGGTGCTGAACAATAGAGTGATCGGCGAGGGCTGGAACAGAGCCATCGGCCTGCACGACCCTACAGCACACGCAGAGATCATGGCACTGAGGCAGGGAGGCCTGGTCATGCAGAATTACCGCCTGATCGATGCCACCCTGTATGTGACATTCGAGCCATGCGTGATGTGCGCAGGAGCAATGATCCACAGCAGGATCGGCCGCGTGGTGTTTGGATGGAGGAACTCCAAGAGGGGAGCAGCAGGCTCTCTGATGAACGTGCTGAATTACCCAGGCATGAATCACCGGGTGGAGATCACCGAGGGCATCCTGGCAGACGAGTGCGCCGCCCTGCTGTGCGATTTCTATCGGATGCCCAGACAGGTGTTTAACGCCCAGAAGAAGGCCCAGAGCAGCATCAACTCCGGAGGATCTAGCGGAGGCTCCTCTGGCTCTGAGACACCTGGCACAAGCGAGAGCGCAACACCTGAAAGCAGCGGGGGCAGCAGCGGGGGATCCGACAAGAAGTACAGCATCGGCCTGGCCATCGGCACCAACTCTGTGGGCTGGGCCGTGATCACCGACGAGTACAAGGTGCCCAGCAAGAAATTCAAGGTGCTGGGCAACACCGACCGGCACAGCATCAAGAAGAACCTGATCGGAGCCCTGCTGTTCGACAGCGGCGAAACAGCCGAGGCCACCCGGCTGAAGAGAACCGCCAGAAGAAGATACACCAGACGGAAGAACCGGATCTGCTATCTGCAAGAGATCTTCAGCAACGAGATGGCCAAGGTGGACGACAGCTTCTTCCACAGACTGGAAGAGTCCTTCCTGGTGGAAGAGGATAAGAAGCACGAGCGGCACCCCATCTTCGGCAACATCGTGGACGAGGTGGCCTACCACGAGAAGTACCCCACCATCTACCACCTGAGAAAGAAACTGGTGGACAGCACCGACAAGGCCGACCTGCGGCTGATCTATCTGGCCCTGGCCCACATGATCAAGTTCCGGGGCCACTTCCTGATCGAGGGCGACCTGAACCCCGACAACAGCGACGTGGACAAGCTGTTCATCCAGCTGGTGCAGACCTACAACCAGCTGTTCGAGGAAAACCCCATCAACGCCAGCGGCGTGGACGCCAAGGCCATCCTGTCTGCCAGACTGAGCAAGAGCAGACGGCTGGAAAATCTGATCGCCCAGCTGCCCGGCGAGAAGAAGAATGGCCTGTTCGGAAACCTGATTGCCCTGAGCCTGGGCCTGACCCCCAACTTCAAGAGCAACTTCGACCTGGCCGAGGATGCCAAACTGCAGCTGAGCAAGGACACCTACGACGACGACCTGGACAACCTGCTGGCCCAGATCGGCGACCAGTACGCCGACCTGTTTCTGGCCGCCAAGAACCTGTCCGACGCCATCCTGCTGAGCGACATCCTGAGAGTGAACACCGAGATCACCAAGGCCCCCCTGAGCGCCTCTATGATCAAGAGATACGACGAGCACCACCAGGACCTGACCCTGCTGAAAGCTCTCGTGCGGCAGCAGCTGCCTGAGAAGTACAAAGAGATTTTCTTCGACCAGAGCAAGAACGGCTACGCCGGCTACATTGACGGCGGAGCCAGCCAGGAAGAGTTCTACAAGTTCATCAAGCCCATCCTGGAAAAGATGGACGGCACCGAGGAACTGCTCGTGAAGCTGAACAGAGAGGACCTGCTGCGGAAGCAGCGGACCTTCGACAACGGCAGCATCCCCCACCAGATCCACCTGGGAGAGCTGCACGCCATTCTGCGGCGGCAGGAAGATTTTTACCCATTCCTGAAGGACAACCGGGAAAAGATCGAGAAGATCCTGACCTTCCGCATCCCCTACTACGTGGGCCCTCTGGCCAGGGGAAACAGCAGATTCGCCTGGATGACCAGAAAGAGCGAGGAAACCATCACCCCCTGGAACTTCGAGGAAGTGGTGGACAAGGGCGCTTCCGCCCAGAGCTTCATCGAGCGGATGACCAACTTCGATAAGAACCTGCCCAACGAGAAGGTGCTGCCCAAGCACAGCCTGCTGTACGAGTACTTCACCGTGTATAACGAGCTGACCAAAGTGAAATACGTGACCGAGGGAATGAGAAAGCCCGCCTTCCTGAGCGGCGAGCAGAAAAAGGCCATCGTGGACCTGCTGTTCAAGACCAACCGGAAAGTGACCGTGAAGCAGCTGAAAGAGGACTACTTCAAGAAAATCGAGTGCTTCGACTCCGTGGAAATCTCCGGCGTGGAAGATCGGTTCAACGCCTCCCTGGGCACATACCACGATCTGCTGAAAATTATCAAGGACAAGGACTTCCTGGACAATGAGGAAAACGAGGACATTCTGGAAGATATCGTGCTGACCCTGACACTGTTTGAGGACAGAGAGATGATCGAGGAACGGCTGAAAACCTATGCCCACCTGTTCGACGACAAAGTGATGAAGCAGCTGAAGCGGCGGAGATACACCGGCTGG |
| GGCAGGCTGAGCCGGAAGCTGATCAACGGCATCCGGGACAAGCAGTCCGGCAAGACAATCCTGGATTTCCTGAAGTCCGACGGCTTCGCCAACAGAAACTTCATGCAGCTGATCCACGACGACAGCCTGACCTTTAAAGAGGACATCCAGAAAGCCCAGGTGTCCGGCCAGGGCGATAGCCTGCACGAGCACATTGCCAATCTGGCCGGCAGCCCCGCCATTAAGAAGGGCATCCTGCAGACAGTGAAGGTGGTGGACGAGCTCGTGAAAGTGATGGGCCGGCACAAGCCCGAGAACATCGTGATCGAAATGGCCAGAGAGAACCAGACCACCCAGAAGGGACAGAAGAACAGCCGCGAGAGAATGAAGCGGATCGAAGAGGGCATCAAAGAGCTGGGCAGCCAGATCCTGAAAGAACACCCCGTGGAAAACACCCAGCTGCAGAACGAGAAGCTGTACCTGTACTACCTGCAGAATGGGCGGGATATGTACGTGGACCAGGAACTGGACATCAACCGGCTGTCCGACTACGATGTGGACCATATCGTGCCTCAGAGCTTTCTGAAGGACGACTCCATCGACAACAAGGTGCTGACCAGAAGCGACAAGAACCGGGGCAAGAGCGACAACGTGCCCTCCGAAGAGGTCGTGAAGAAGATGAAGAACTACTGGCGGCAGCTGCTGAACGCCAAGCTGATTACCCAGAGAAAGTTCGACAATCTGACCAAGGCCGAGAGAGGCGGCCTGAGCGAACTGGATAAGGCCGGCTTCATCAAGAGACAGCTGGTGGAAACCCGGCAGATCACAAAGCACGTGGCACAGATCCTGGACTCCCGGATGAACACTAAGTACGACGAGAATGACAAGCTGATCCGGGAAGTGAAAGTGATCACCCTGAAGTCCAAGCTGGTGTCCGATTTCCGGAAGGATTTCCAGTTTTACAAAGTGCGCGAGATCAACAACTACCACCACGCCCACGACGCCTACCTGAACGCCGTCGTGGGAACCGCCCTGATCAAAAAGTACCCTAAGCTGGAAAGCGAGTTCGTGTACGGCGACTACAAGGTGTACGACGTGCGGAAGATGATCGCCAAGAGCGAGCAGGAAATCGGCAAGGCTACCGCCAAGTACTTCTTCTACAGCAACATCATGAACTTTTTCAAGACCGAGATTACCCTGGCCAACGGCGAGATCCGGAAGCGGCCTCTGATCGAGACAAACGGCGAAACCGGGGAGATCGTGTGGGATAAGGGCCGGGATTTTGCCACCGTGCGGAAAGTGCTGAGCATGCCCCAAGTGAATATCGTGAAAAAGACCGAGGTGCAGACAGGCGGCTTCAGCAAAGAGTCTATCCTGCCCAAGAGGAACAGCGATAAGCTGATCGCCAGAAAGAAGGACTGGGACCCTAAGAAGTACGGCGGCTTCGACAGCCCCACCGTGGCCTATTCTGTGCTGGTGGTGGCCAAAGTGGAAAAGGGCAAGTCCAAGAAACTGAAGAGTGTGAAAGAGCTGCTGGGGATCACCATCATGGAAAGAAGCAGCTTCGAGAAGAATCCCATCGACTTTCTGGAAGCCAAGGGCTACAAAGAAGTGAAAAAGGACCTGATCATCAAGCTGCCTAAGTACTCCCTGTTCGAGCTGGAAAACGGCCGGAAGAGAATGCTGGCCTCTGCCGGCGAACTGCAGAAGGGAAACGAACTGGCCCTGCCCTCCAAATATGTGAACTTCCTGTACCTGGCCAGCCACTATGAGAAGCTGAAGGGCTCCCCCGAGGATAATGAGCAGAAACAGCTGTTTGTGGAACAGCACAAGCACTACCTGGACGAGATCATCGAGCAGATCAGCGAGTTCTCCAAGAGAGTGATCCTGGCCGACGCTAATCTGGACAAAGTGCTGTCCGCCTACAACAAGCACCGGGATAAGCCCATCAGAGAGCAGGCCGAGAATATCATCCACCTGTTTACCCTGACCAATCTGGGAGCCCCTGCCGCCTTCAAGTACTTTGACACCACCATCGACCGGAAGAGGTACACCAGCACCAAAGAGGTGCTGGACGCCACCCTGATCCACCAGAGCATCACCGGCCTGTACGAGACACGGATCGACCTGTCTCAGCTGGGAGGCGACAAAAGAACCGCCGACGGCAGCGAATTCGAGCCCAAGAAGAAGAGGAAAGTCTCAGGGGGAAGTGGTGGTTCTGGTGGATCGGTGACCCCAGCTCTCCAGATGAAGAAGCCGAAGCAATTCTGTAGAAGGATGGGCCAGAAAAAGCAGAGGCCGGCCAGGGCTGGGCAACCTCATTCCTCGTCCGATGCGGCACAAGCCCCAGCAGAACAGCCTCATTCTTCCTCTGACGCCGCCCAAGCGCCCTGTCCACGTGAAAGATGTCTCGGACCTCCTACCACGCCAGGTCCATACCGCAGCATCTACTTTAGCTCGCCCAAGGGCCATTTGACGAGGTTGGGGCTGGAGTTCTTTGATCAACCGGCCGTTCCACTCGCAAGAGCGTTCTTGGGGCAAGTGCTGGTTAGGAGGTTGCCTAACGGAACGGAATTGCGCGGACGCATCGTTGAGACAGAGGCCTACCTGGGTCCTGAGGATGAAGCTGCGCATAGCCGGGGAGGCAGGCAAACACCTAGGAATAGAGGAATGTTCATGAAGCCAGGGACGCTGTACGTCTACATAATATACCGCATGTACTTTTGTATGTCGATCAGCAGTCAGGGCGACGGAGCGTGCGTTCTTCTTAGAGCCCTCGAGCCTCTGGAAGGCCTGGAGACGATGAGACAGCTTAGGGCCACATTGCGGGCTGCTACTGCTGCAAGGGTACTCGCGGACCGTGAACTCTGTTCTGGTCCTTCCAAGCTGTGCCAGGCATTGGCCATAAATAAGAGCTTTGACCAGAGAGACCTCGCTCAGGACGAAGCAGTTTGGCTCGAACGCGGACCCCTGGAACCAAGCGAACCAGCTGTTGTTGCAGCAGCGAGAGTTGGAGTGGGTCATGCGGGAGAATGGGCCAGGAAACCCCTGCGTTTTTATGTACGTGGGTCACCGTGGGTGTCAGTGGTTGATCGCGTCGCCGAACAGGATACTCAAGCGAGTGGAGGTTCGAAACGGACAGCAGACGGAAGCGAGTTTGAGCCGAAGAAGAAAAGAAAAGTGATGCCGGTGGCGAGGCCGGAGCCGCAGGAGCCGCGGGTGATCGCCCATGTCGACATGGATTGCTTCTACGTCCAAGTTGAGCAGCGGAGGAACCCGGAGCTCAGGGGGCAGCCGACCGCCGTGGTGCAGTACAACGACTGGAAAGGCGGCGGGCTGATTGCCGTCAGCTACGAGGCCCGGAAGTTCGGCGTGAAGAGGTCCATGCGTGGTGATGAGGCCAAGATGGTCTGTCCAAGTATCAATTTGGTTCAGGTTCCGGTGGCCCGTGACAAGGCCGATCTTAATGTTTACCGTAGTGCTGGCTCTGAGGTTGTCACAATCCTCTCGACCAAGGGGAAGTGTGAGCGAGCATCCATCGACGAAGTTTATCTTGACCTTACTGATGCAGCCAAGGAAATGCTTTTAGAATCTCCACCGGAGTTATTGGAGTTGATTTTTGAGGAGGCCACAAAGTCAAATATCCTGGGCCTTCCTTCTGATGTCAGCAACAGAGAAGATAGCGTGAGGGCATGGCTTTGTCGAGCCGATGCTGATTACCAGGATAAGTTACTATCTTGTGGGGCTATAATTGTTGCGCAATTACGGGTCAAGGTTTTGGAGGAAACCCAATTCACATGTTCTGCTGGTATTGCTCACAATAAGATGTTAGCAAAACTTGTCAGTGGGATGCACAAACCTGCTCAGCAGACAGTTGTTCCATCATCAGCAGTTCAAGATTTTCTAGTATCACTGCCCATAAAGAAGATGAAGCAACTTGGTGGTAAGCTTGGGAGTTCCTTGCAGGATGACCTTGGGGTCAATACTGTTGGTGATCTTCTAAGTTTTACAGAGGACAAATTACAAGAGTACTATGGAGTAAATACAGGGACGTGGTTATGGAAGATTGCAAGAGGTATTAGTGGAGAAGAAGTTGAGGATCGTCTTCTACCAAAGAGCCATGGGTGTGGAAAGACATTTCCTGGCCCAAAAGCATTAAAGAATAATGCTTCTGTTAAGACATGGCTGGATCGACTTTGTGAAGAACTGAGCGAACGGATTCAGTCTGATTTGAACCAGAATAAGAGAATTGCTCAAACACTAACTCTCTATGCCAGGGCATGTAAGAAAAACAAGTCTGATTCAATCAAGAAATTTCCTTCCAAATCTTGTCCGTTGCGTTATGGGACTGTCAAAATTCAAGAAGATGCAATGAAGCTATTTGATCTGGGCTTCATGATTTCTTGGGTTCTCAGAACACAAAATGGAGCATAACATCTCTTTCTGTTTCAGCAAGCAAAATATTCGACATACCAATTGGAACAAGCTCTATCTTGAGATACATTAAAGGACCAAATTCCACTGTTTCACCAGCTAACCTCGATTGTTCTTCTTTACCTGAGGATCCATCTCTTGGTAACAAGTTATATATAGCACCTAATCATGAAGAACACTGTGAACCATCATTATCTGAGAAAGAAGACTATGGAAACAATAGTAATTTAGCCAAACAGTGTCAAATAAAGGAAGAAAAAAAGGTGTCAAAGAAGTTAACTGAAGTTAAGGGAACTTGCTCTATCTGAAATTTCTTTCACAAAGTCCTGTACTCAGTGAGAAAAGAAAAATTGACAGCTTAATTTGCAGTCATCCAGGTCCTGAAAGTTCTTCAGAACCGAATAAAGCTGAAGAACACAAGGCTGCGCAGTATGTTGATAGGAACAAGTTTAACACTGCTGGGAGTAACTCTGCCAGCAGTAGCACATGGATGTTCAACGTTGAAGATATCGATCCAGCCGTAGTGGAAGAACTGCCGCCGGAAATACAAAGAGAAATACATGGATGGATTCGCCCGCCAAAGCAATCAAGCTCAAAGACACGGGGTTCTACCATTTCGTCTTACTTCCAACCTGCAAAGAGGTCAGGATAGAAAAAGCCTGAACTCACCGCGACGTCTGTCGAGAAGTTTCTGATCGAAAAGTTCGACAGCGTCTCCGACCTGATGCAGCTCTCGGAGGGCGAAGAATCTCGTGCTTTCAGCTTCGATGTAGGAGGGCGTGGATATGTCCTGCGGGTAAATAGCTGCGCCGATGGTTTCTACAAAGATCGTTATGTTTATCGGCACTTTGCATCGGCCGCGCTCCCGATTCCGGAAGTGCTTGACATTGGGGAGTTTAGCGAGAGCCTGACCTATTGCATCTCCCGCCGTGCACAGGGTGTCACGTTGCAAGACCTGCCTGAAACCGAACTGCCCGCTGTTCTACAACCGGTCGCGGAGGCTATGGATGCGATCGCTGCGGCCGATCTTAGCCAGACGAGCGGGTTCGGCCCATTCGGACCGCAAGGAATCGGTCAATACACTACATGGCGTGATTTCATATGCGCGATTGCTGATCCCCATGTGTATCACTGGCAAACTGTGATGGACGACACCGTCAGTGCGTCCGTCGCGCAGGCTCTCGATGAGCTGATGCTTTGGGCCGAGGACTGCCCCGAAGTCCGGCACCTCGTGCACGCGGATTTCGGCTCCAACAATGTCCTGACGGACAATGGCCGCATAACAGCGGTCATTGACTGGAGCGAGGCGATGTTCGGGGATTCCCAATACGAGGTCGCCAACATCTTCTTCTGGAGGCCGTGGTTGGCTTGTATGGAGCAGCAGACGCGCTACTTCGAGCGGAGGCATCCGGAGCTTGCAGGATCGCCACGACTCCGGGCGTATATGCTCCGCATTGGTCTTGACCAACTCTATCAGAGCTTGGTTGACGGCAATTTCGATGATGCAGCTTGGGCGCAGGGTCGATGCGACGCAATCGTCCGATCCGGAGCCGGGACTGTCGGGCGTACACAAATCGCCCGCAGAAGCGCGGCCGTCTGGACCGATGGCTGTGTAGAAGTACTCGCCGATAGTGGAAACCGACGCCCCAGCACTCGTCCGAGGGCAAAGAAATAG |
| >Danshen TLS polymerase η  ATGCCGGTGGCGAGGCCGGAGCCGCAGGAGCCGCGGGTGATCGCCCATGTCGACATGGATTGCTTCTACGTCCAAGTTGAGCAGCGGAGGAACCCGGAGCTCAGGGGGCAGCCGACCGCCGTGGTGCAGTACAACGACTGGAAAGGCGGCGGGCTGATTGCCGTCAGCTACGAGGCCCGGAAGTTCGGCGTGAAGAGGTCCATGCGTGGTGATGAGGCCAAGATGGTCTGTCCAAGTATCAATTTGGTTCAGGTTCCGGTGGCCCGTGACAAGGCCGATCTTAATGTTTACCGTAGTGCTGGCTCTGAGGTTGTCACAATCCTCTCGACCAAGGGGAAGTGTGAGCGAGCATCCATCGACGAAGTTTATCTTGACCTTACTGATGCAGCCAAGGAAATGCTTTTAGAATCTCCACCGGAGTTATTGGAGTTGATTTTTGAGGAGGCCACAAAGTCAAATATCCTGGGCCTTCCTTCTGATGTCAGCAACAGAGAAGATAGCGTGAGGGCATGGCTTTGTCGAGCCGATGCTGATTACCAGGATAAGTTACTATCTTGTGGGGCTATAATTGTTGCGCAATTACGGGTCAAGGTTTTGGAGGAAACCCAATTCACATGTTCTGCTGGTATTGCTCACAATAAGATGTTAGCAAAACTTGTCAGTGGGATGCACAAACCTGCTCAGCAGACAGTTGTTCCATCATCAGCAGTTCAAGATTTTCTAGTATCACTGCCCATAAAGAAGATGAAGCAACTTGGTGGTAAGCTTGGGAGTTCCTTGCAGGATGACCTTGGGGTCAATACTGTTGGTGATCTTCTAAGTTTTACAGAGGACAAATTACAAGAGTACTATGGAGTAAATACAGGGACGTGGTTATGGAAGATTGCAAGAGGTATTAGTGGAGAAGAAGTTGAGGATCGTCTTCTACCAAAGAGCCATGGGTGTGGAAAGACATTTCCTGGCCCAAAAGCATTAAAGAATAATGCTTCTGTTAAGACATGGCTGGATCGACTTTGTGAAGAACTGAGCGAACGGATTCAGTCTGATTTGAACCAGAATAAGAGAATTGCTCAAACACTAACTCTCTATGCCAGGGCATGTAAGAAAAACAAGTCTGATTCAATCAAGAAATTTCCTTCCAAATCTTGTCCGTTGCGTTATGGGACTGTCAAAATTCAAGAAGATGCAATGAAGCTATTTGATCTGGGCTTCATGATTTCTTGGGTTCTCAGAACACAAAATGGAGCATAACATCTCTTTCTGTTTCAGCAAGCAAAATATTCGACATACCAATTGGAACAAGCTCTATCTTGAGATACATTAAAGGACCAAATTCCACTGTTTCACCAGCTAACCTCGATTGTTCTTCTTTACCTGAGGATCCATCTCTTGGTAACAAGTTATATATAGCACCTAATCATGAAGAACACTGTGAACCATCATTATCTGAGAAAGAAGACTATGGAAACAATAGTAATTTAGCCAAACAGTGTCAAATAAAGGAAGAAAAAAAGGTGTCAAAGAAGTTAACTGAAGTTAAGGGAACTTGCTCTATCTGAAATTTCTTTCACAAAGTCCTGTACTCAGTGAGAAAAGAAAAATTGACAGCTTAATTTGCAGTCATCCAGGTCCTGAAAGTTCTTCAGAACCGAATAAAGCTGAAGAACACAAGGCTGCGCAGTATGTTGATAGGAACAAGTTTAACACTGCTGGGAGTAACTCTGCCAGCAGTAGCACATGGATGTTCAACGTTGAAGATATCGATCCAGCCGTAGTGGAAGAACTGCCGCCGGAAATACAAAGAGAAATACATGGATGGATTCGCCCGCCAAAGCAATCAAGCTCAAAGACACGGGGTTCTACCATTTCGTCTTACTTCCAACCTGCAAAGAGGTCAGGATAG |
| >Sm CBEmax01/02/03, NLS-Anc689(R33A)-linker-nCas9-UGI-NLS AGCAGTGAAACCGGACCAGTGGCAGTGGACCCAACCCTGAGGAGACGGATTGAGCCCCATGAATTTGAAGTGTTCTTTGACCCAAGGGAGCTGAGGAAGGAGACATGCCTGCTGTACGAGATCAAGTGGGGCACAAGCCACAAGATCTGGCGCCACAGCTCCAAGAACACCACAAAGCACGTGGAAGTGAATTTCATCGAGAAGTTTACCTCCGAGCGGCACTTCTGCCCCTCTACCAGCTGTTCCATCACATGGTTTCTGTCTTGGAGCCCTTGCGGCGAGTGTTCCAAGGCCATCACCGAGTTCCTGTCTCAGCACCCTAACGTGACCCTGGTCATCTACGTGGCCCGGCTGTATCACCACATGGACCAGCAGAACAGGCAGGGCCTGCGCGATCTGGTGAATTCTGGCGTGACCATCCAGATCATGACAGCCCCAGAGTACGACTATTGCTGGCGGAACTTCGTGAATTATCCACCTGGCAAGGAGGCACACTGGCCAAGATACCCACCCCTGTGGATGAAGCTGTATGCACTGGAGCTGCACGCAGGAATCCTGGGCCTGCCTCCATGTCTGAATATCCTGCGGAGAAAGCAGCCCCAGCTGACATTTTTCACCATTGCTCTGCAGTCTTGTCACTATCAGCGGCTGCCTCCTCATATTCTGTGGGCTACAGGCCTTAAAGACAAGAAGTACAGCATCGGCCTGGCCATCGGCACCAACTCTGTGGGCTGGGCCGTGATCACCGACGAGTACAAGGTGCCCAGCAAGAAATTCAAGGTGCTGGGCAACACCGACCGGCACAGCATCAAGAAGAACCTGATCGGAGCCCTGCTGTTCGACAGCGGCGAAACAGCCGAGGCCACCCGGCTGAAGAGAACCGCCAGAAGAAGATACACCAGACGGAAGAACCGGATCTGCTATCTGCAAGAGATCTTCAGCAACGAGATGGCCAAGGTGGACGACAGCTTCTTCCACAGACTGGAAGAGTCCTTCCTGGTGGAAGAGGATAAGAAGCACGAGCGGCACCCCATCTTCGGCAACATCGTGGACGAGGTGGCCTACCACGAGAAGTACCCCACCATCTACCACCTGAGAAAGAAACTGGTGGACAGCACCGACAAGGCCGACCTGCGGCTGATCTATCTGGCCCTGGCCCACATGATCAAGTTCCGGGGCCACTTCCTGATCGAGGGCGACCTGAACCCCGACAACAGCGACGTGGACAAGCTGTTCATCCAGCTGGTGCAGACCTACAACCAGCTGTTCGAGGAAAACCCCATCAACGCCAGCGGCGTGGACGCCAAGGCCATCCTGTCTGCCAGACTGAGCAAGAGCAGACGGCTGGAAAATCTGATCGCCCAGCTGCCCGGCGAGAAGAAGAATGGCCTGTTCGGAAACCTGATTGCCCTGAGCCTGGGCCTGACCCCCAACTTCAAGAGCAACTTCGACCTGGCCGAGGATGCCAAACTGCAGCTGAGCAAGGACACCTACGACGACGACCTGGACAACCTGCTGGCCCAGATCGGCGACCAGTACGCCGACCTGTTTCTGGCCGCCAAGAACCTGTCCGACGCCATCCTGCTGAGCGACATCCTGAGAGTGAACACCGAGATCACCAAGGCCCCCCTGAGCGCCTCTATGATCAAGAGATACGACGAGCACCACCAGGACCTGACCCTGCTGAAAGCTCTCGTGCGGCAGCAGCTGCCTGAGAAGTACAAAGAGATTTTCTTCGACCAGAGCAAGAACGGCTACGCCGGCTACATTGACGGCGGAGCCAGCCAGGAAGAGTTCTACAAGTTCATCAAGCCCATCCTGGAAAAGATGGACGGCACCGAGGAACTGCTCGTGAAGCTGAACAGAGAGGACCTGCTGCGGAAGCAGCGGACCTTCGACAACGGCAGCATCCCCCACCAGATCCACCTGGGAGAGCTGCACGCCATTCTGCGGCGGCAGGAAGATTTTTACCCATTCCTGAAGGACAACCGGGAAAAGATCGAGAAGATCCTGACCTTCCGCATCCCCTACTACGTGGGCCCTCTGGCCAGGGGAAACAGCAGATTCGCCTGGATGACCAGAAAGAGCGAGGAAACCATCACCCCCTGGAACTTCGAGGAAGTGGTGGACAAGGGCGCTTCCGCCCAGAGCTTCATCGAGCGGATGACCAACTTCGATAAGAACCTGCCCAACGAGAAGGTGCTGCCCAAGCACAGCCTGCTGTACGAGTACTTCACCGTGTATAACGAGCTGACCAAAGTGAAATACGTGACCGAGGGAATGAGAAAGCCCGCCTTCCTGAGCGGCGAGCAGAAAAAGGCCATCGTGGACCTGCTGTTCAAGACCAACCGGAAAGTGACCGTGAAGCAGCTGAAAGAGGACTACTTCAAGAAAATCGAGTGCTTCGACTCCGTGGAAATCTCCGGCGTGGAAGATCGGTTCAACGCCTCCCTGGGCACATACCACGATCTGCTGAAAATTATCAAGGACAAGGACTTCCTGGACAATGAGGAAAACGAGGACATTCTGGAAGATATCGTGCTGACCCTGACACTGTTTGAGGACAGAGAGATGATCGAGGAACGGCTGAAAACCTATGCCCACCTGTTCGACGACAAAGTGATGAAGCAGCTGAAGCGGCGGAGATACACCGGCTGGGGCAGGCTGAGCCGGAAGCTGATCAACGGCATCCGGGACAAGCAGTCCGGCAAGACAATCCTGGATTTCCTGAAGTCCGACGGCTTCGCCAACAGAAACTTCATGCAGCTGATCCACGACGACAGCCTGACCTTTAAAGAGGACATCCAGAAAGCCCAGGTGTCCGGCCAGGGCGATAGCCTGCACGAGCACATTGCCAATCTGGCCGGCAGCCCCGCCATTAAGAAGGGCATCCTGCAGACAGTGAAGGTGGTGGACGAGCTCGTGAAAGTGATGGGCCGGCACAAGCCCGAGAACATCGTGATCGAAATGGCCAGAGAGAACCAGACCACCCAGAAGGGACAGAAGAACAGCCGCGAGAGAATGAAGCGGATCGAAGAGGGCATCAAAGAGCTGGGCAGCCAGATCCTGAAAGAACACCCCGTGGAAAACACCCAGCTGCAGAACGAGAAGCTGTACCTGTACTACCTGCAGAATGGGCGGGATATGTACGTGGACCAGGAACTGGACATCAACCGGCTGTCCGACTACGATGTGGACCATATCGTGCCTCAGAGCTTTCTGAAGGACGACTCCATCGACAACAAGGTGCTGACCAGAAGCGACAAGAACCGGGGCAAGAGCGACAACGTGCCCTCCGAAGAGGTCGTGAAGAAGATGAAGAACTACTGGCGGCAGCTGCTGAACGCCAAGCTGATTACCCAGAGAAAGTTCGACAATCTGACCAAGGCCGAGAGAGGCGGCCTGAGCGAACTGGATAAGGCCGGCTTCATCAAGAGACAGCTGGTGGAAACCCGGCAGATCACAAAGCACGTGGCACAGATCCTGGACTCCCGGATGAACACTAAGTACGACGAGAATGACAAGCTGATCCGGGAAGTGAAAGTGATCACCCTGAAGTCCAAGCTGGTGTCCGATTTCCGGAAGGATTTCCAGTTTTACAAAGTGCGCGAGATCAACAACTACCACCACGCCCACGACGCCTACCTGAACGCCGTCGTGGGAACCGCCCTGATCAAAAAGTACCCTAAGCTGGAAAGCGAGTTCGTGTACGGCGACTACAAGGTGTACGACGTGCGGAAGATGATCGCCAAGAGCGAGCAGGAAATCGGCAAGGCTACCGCCAAGTACTTCTTCTACAGCAACATCATGAACTTTTTCAAGACCGAGATTACCCTGGCCAACGGCGAGATCCGGAAGCGGCCTCTGATCGAGACAAACGGCGAAACCGGGGAGATCGTGTGGGATAAGGGCCGGGATTTTGCCACCGTGCGGAAAGTGCTGAGCATGCCCCAAGTGAATATCGTGAAAAAGACCGAGGTGCAGACAGGCGGCTTCAGCAAAGAGTCTATCCTGCCCAAGAGGAACAGCGATAAGCTGATCGCCAGAAAGAAGGACTGGGACCCTAAGAAGTACGGCGGCTTCGACAGCCCCACCGTGGCCTATTCTGTGCTGGTGGTGGCCAAAGTGGAAAAGGGCAAGTCCAAGAAACTGAAGAGTGTGAAAGAGCTGCTGGGGATCACCATCATGGAAAGAAGCAGCTTCGAGAAGAATCCCATCGACTTTCTGGAAGCCAAGGGCTACAAAGAAGTGAAAAAGGACCTGATCATCAAGCTGCCTAAGTACTCCCTGTTCGAGCTGGAAAACGGCCGGAAGAGAATGCTGGCCTCTGCCGGCGAACTGCAGAAGGGAAACGAACTGGCCCTGCCCTCCAAATATGTGAACTTCCTGTACCTGGCCAGCCACTATGAGAAGCTGAAGGGCTCCCCCGAGGATAATGAGCAGAAACAGCTGTTTGTGGAACAGCACAAGCACTACCTGGACGAGATCATCGAGCAGATCAGCGAGTTCTCCAAGAGAGTGATCCTGGCCGACGCTAATCTGGACAAAGTGCTGTCCGCCTACAACAAGCACCGGGATAAGCCCATCAGAGAGCAGGCCGAGAATATCATCCACCTGTTTACCCTGACCAATCTGGGAGCCCCTGCCGCCTTCAAGTACTTTGACACCACCATCGACCGGAAGAGGTACACCAGCACCAAAGAGGTGCTGGACGCCACCCTGATCCACCAGAGCATCACCGGCCTGTACGAGACACGGATCGACCTGTCTCAGCTGGGAGGCGACAGCGGCGGGAGCGGCGGGAGCGGCGGGAGCGGGGGGAGCACTAATCTGAGCGACATCATTGAGAAGGAGACTGGGAAACAGCTGGTCATTCAGGAGTCCATCCTGATGCTGCCTGAGGAGGTGGAGGAAGTGATCGGCAACAAGCCAGAGTCTGACATCCTGGTGCACACCGCCTACGACGAGTCCACAGATGAGAATGTGATGCTGCTGACCTCTGACGCCCCCGAGTATAAGCCTTGGGCCCTGGTCATCCAGGATTCTAACGGCGAGAATAAGATCAAGATGCTGAGCGGAGGCTCCGGAGGATCTGGAGGCAGCACCAACCTGTCTGACATCATCGAGAAGGAGACAGGCAAGCAGCTGGTCATCCAGGAGAGCATCCTGATGCTGCCCGAAGAAGTCGAAGAAGTGATCGGAAACAAGCCTGAGAGCGATATCCTGGTCCATACCGCCTACGACGAGAGTACCGACGAAAATGTGATGCTGCTGACATCCGACGCCCCAGAGTATAAGCCCTGGGCTCTGGTCATCCAGGATTCCAACGGAGAGAACAAAATCAAAATGCTGTCTGGCGGCTCAAAAAGAACCGCCGACGGCAGCGAATTCGAGCCCAAGAAGAAGAGGAAAGTC |

**Supplementary Table 5.** Gene accession numbers in this study.

| **Locus** | **Accession number (Gene)** | **Accession number (Protein)** |
| --- | --- | --- |
| *SmMYB36* | LOC131015177 | XP_057799436.1 |
| *SmMYB39* | LOC131025187 | XP_057810813.1 |
| *SmMYB1* | LOC131016653 | XP_057801337.1 |
| *SmMYB76* | LOC131001051 | XP_057783206.1 |
| *SmJAZ3* | LOC131019618 | XP_057804193.1 |
| *SmbHLH3* | LOC131007779 | XP_057790679.1 |
| *SmbZIP2* | LOC131010826 | XP_057794474.1 |
| *SmC4H* | LOC130997738 | XP_057779129.1" |
| *SmKSL2* | KC814643.1 | AHJ59325.1 |
| *SmHMGR1* | EU680958.1 | ACD37361.1 |
| *SmCPS2* | JN831114.1 | AEZ55684.1 |
